# Differential distribution of Y-chromosome haplotypes in Swiss and Southern European goat breeds

**DOI:** 10.1038/s41598-017-15593-1

**Published:** 2017-11-23

**Authors:** Oriol Vidal, Cord Drögemüller, Gabriela Obexer-Ruff, Irene Reber, Jordi Jordana, Amparo Martínez, Valentin Adrian Bâlteanu, Juan Vicente Delgado, Shahin Eghbalsaied, Vincenzo Landi, Felix Goyache, Amadou Traoré, Michele Pazzola, Giuseppe Massimo Vacca, Bouabid Badaoui, Fabio Pilla, Mariasilvia D’Andrea, Isabel Álvarez, Juan Capote, Abdoallah Sharaf, Àgueda Pons, Marcel Amills

**Affiliations:** 1 Departament de Biologia, Universitat de Girona, 17003 Girona, Spain; 2 Institute of Genetics, University of Bern, Bern, 3001 Switzerland; 3 Departament de Ciència Animal i dels Aliments, Facultat de Veterinària, Universitat Autònoma de Barcelona, 08193 Bellaterra, Spain; 4 Departamento de Genética, Universidad de Córdoba, 14071 Córdoba, Spain; 5 Institute of Life Sciences, Faculty of Animal Science and Biotechnologies, University of Agricultural Sciences and Veterinary Medicine, 400372 Cluj-Napoca, Romania; 6 Transgenesis Center of Excellence, Isfahan (Khorasgan) Branch, Islamic Azad University, Isfahan, Iran; 7 Área de Genética y Reproducción Animal, SERIDA-Deva, Camino de Rioseco 1225, Gijón, 33394 Spain; 8 Institut de l’Environnement et Recherches Agricoles, 04 BP 8645, Ouagadougou, 04 Burkina Faso; 9 Department of Veterinary Medicine, University of Sassari, 07100 Sassari, Italy; 10 University Mohammed V, Agdal, Faculty of Sciences, 4 Av. Ibn Battota, Rabat, Morocco; 11 Dipartimento Agricoltura, Ambiente e Alimenti, Università Degli Studi Del Molise, Campobasso, Italy; 12 Instituto Canario de Investigaciones Agrarias, Canary Islands, Tenerife, La Laguna 38108 Spain; 13 Genetic Department, Faculty of Agriculture, Ain Shams University, Cairo, 11241 Egypt; 14 Institute of Parasitology, Biology Centre, Czech Academy of Sciences, 37005 České Budějovice, Czechia; 15 Department of Animal Genetics, Center for Research in Agricultural Genomics (CRAG), CSIC-IRTA-UAB-UB, Campus Universitat Autònoma de Barcelona, Bellaterra, 08193 Spain; 16 Unitat de Races Autòctones, Servei de Millora Agrària, (SEMILLA-SAU), Son Ferriol, 07198 Spain

## Abstract

The analysis of Y-chromosome variation has provided valuable clues about the paternal history of domestic animal populations. The main goal of the current work was to characterize Y-chromosome diversity in 31 goat populations from Central Eastern (Switzerland and Romania) and Southern Europe (Spain and Italy) as well as in reference populations from Africa and the Near East. Towards this end, we have genotyped seven single nucleotide polymorphisms (SNPs), mapping to the *SRY, ZFY, AMELY* and *DDX3Y* Y-linked loci, in 275 bucks from 31 populations. We have observed a low level of variability in the goat Y-chromosome, with just five haplotypes segregating in the whole set of populations. We have also found that Swiss bucks carry exclusively Y1 haplotypes (Y1A: 24%, Y1B1: 15%, Y1B2: 43% and Y1C: 18%), while in Italian and Spanish bucks Y2A is the most abundant haplotype (77%). Interestingly, in Carpathian goats from Romania the Y2A haplotype is also frequent (42%). The high Y-chromosome differentiation between Swiss and Italian/Spanish breeds might be due to the post-domestication spread of two different Near Eastern genetic stocks through the Danubian and Mediterranean corridors. Historical gene flow between Southern European and Northern African goats might have also contributed to generate such pattern of genetic differentiation.

## Introduction

Because of its male-limited transmission and lack of recombination^1^, Y-chromosome variation provides a simple and highly informative record of the paternal history of domestic species^2–4^. In general, Y-chromosome diversity is quite low due to small effective size (*e.g*. high variance in male reproductive success) and low mutation rate combined with the erosive effects of positive and purifying selection on diversity^5^. A first glimpse of caprine Y-chromosome variation was provided by Pidancier *et al*. (2006)^6^, who sequenced fragments of the amelogenin, Y-Linked (*AMELY*) and zinc finger protein, Y-Linked (*ZFY*) genes in wild and domestic goats. By doing so, they defined two common haplotypes C1 and C2 and a third rarer haplotype named C3. Further studies made possible to establish the existence of six Y-chromosome haplotypes *i.e*. Y1A, Y1B, Y1C, Y2A, Y2B, and Y2C^7–10^. Contrary to mitochondrial DNA, the analysis of caprine Y-chromosome variation evidenced the existence of a strong population structure in goats.

Waki *et al*.^9^ sequenced a fragment of the 3′UTR of the *SRY* gene in 210 Asian goats and demonstrated that the most frequent haplotype in Asia is Y1A (62%) followed by Y2B (30%). Asian breeds also showed marked differences with regard to Y-chromosome haplotype distribution^9^. Çinar-Kul *et al*.^8^ investigated Y-chromosome diversity in several Turkish goat breeds by partially sequencing the *AMELY*, *ZFY* and *SRY* genes and found that the most frequent haplotype was Y2A followed by Y1A, whilst a new Y2C minority haplotype was also identified. This study was of particular interest because Eastern Anatolia has been shown to be the unique primary centre of goat domestication^11^. Moreover, genotyping of four single nucleotide polymorphisms (SNPs) mapping to the sex determining region Y (SRY) gene in 46 Moroccan and 44 Portuguese bucks evidenced that haplotype frequencies are remarkably similar in both populations (Y2 is more frequent than Y1A and Y1B), suggesting the existence of gene flow between goats from the Iberian Peninsula and the Maghreb^12^.

Although Y-chromosome diversity has been reported in several European caprine breeds^7,10,12^, the number of sampled populations and individuals is still quite limited. Our goal was to overcome this drawback by genotyping seven Y-chromosome SNPs in 275 bucks from Southern Europe (Italy and Spain, N = 106), Central and East Europe (Switzerland and Romania, N = 113), Africa (Egypt, Burkina-Faso and Nigeria, N = 33) and the Near East (Iran and Oman, N = 23).

## Methods

### Sampling and sequencing of five Y-chromosome regions

Blood and hair were collected in 275 bucks belonging to 31 populations from nine different countries (Table 1). These samples came from four main geographic areas *i.e*. Central and East Europe, Southern Europe, Africa and Near East (Table 1). The isolation of genomic DNA was performed as described by Amills *et al*.^4^. Five regions of the *AMELY*, DEAD-box helicase 3, Y-linked (*DDX3Y*), ubiquitously transcribed tetratricopeptide repeat containing, Y-Linked (*UTY*) and *ZFY* genes were amplified by PCR and subsequently sequenced in a set of ten goats belonging to five different breeds (Saanen, Alpine, Murciano-Granadina, Palmera and Tinerfeña) to identify polymorphic sites. We used previously described ovine primers^2^ in four of the five selected regions (Supplementary Table S1 **)**. Polymerase chain reactions were carried out in a final volume of 30 μL containing 1.5 mm MgCl_2_, 200 μM dNTPs, 0.2 μM of each primer, 25 ng genomic DNA and 0.3 U Taq DNA polymerase (Biogen). The thermal profile was 94 °C for 5′ followed by 35 cycles of 94 °C during 30″, 55 °C for 1′ 30″ and 72 °C during 1′ 30″, ending with an extension step of 5′ at 72 °C. Amplicons were purified with the ExoSAP-IT PCR Product Cleanup Reagent (Thermo Fisher Scientific) and sequenced with the BigDye Terminator v3.1 Cycle Sequencing kit (Thermo Fisher Scientific) and the corresponding amplification primers. Sequencing reactions were electrophoresed in an ABI310 Genetic Analyzer equipment (Applied Biosystems). Sequences were visualized with the SeqAnalysis software (Thermo Fisher Scientific) and aligned with the Multalin program^13^.

**Table 1:** Distribution of Y-chromosome haplotypes in 31 goat populations.

Area	BREED	Country	Y1A	Y1B1	Y1B2	Y1C	Y2
Central and East Europe	Alpine	Switzerland	8	2			
	Appenzell				8		
	Camosciata delle Alpi		1				
	Chamois Coloured goat		5				
	Grisons Striped				9		
	Peacock Goat					9	
	Saanen			3	15		
	St Gallen Booted goat		9				
	Toggenburg				8		
	Valais Blackneck			9			
	Verzasca goat					8	
	Carpathian	Romania	10		1		8
	TOTAL (N = 113)		33	14	41	17	8
Southern Europe	Bermeya	Spain					9
	Blanca Andaluza		1				1
	Guadarrama						10
	Malagueña						4
	Murciano Granadina						23
	Mallorquina						10
	Pitiüsa						15
	Blanca de Rasquera						5
	Garganica	Italy		1	1	1	
	Sarda		16		1		5
	Maltese		3				
	TOTAL (N = 106)		20	1	2	1	82
Africa	Djallonke	Burkina Faso					5
	Sahel						9
	African Dwarf Goat	Nigeria					2
	Nigerian						2
	Zaraibi	Egypt					15
	TOTAL (N = 33)		0	0	0	0	33
Near East	Lori-Bakhtyari Goat	Iran	8				2
	Esfahan Goat		12				
	Oman	Oman					1
	TOTAL (N = 23)		20	0	0	0	3

### Generation and analysis of genotypic data

Seven Y-chromosome polymorphisms, two discovered in this study and five reported in the literature^6,7,10^, were selected to be genotyped in a multiplex assay (Table 2). A total of 275 bucks were typed in the Veterinary Service of Molecular Genetics (http://svgm.es/ca/Home) of the Universitat Autònoma de Barcelona. Genomic DNA samples were distributed in 384-well sample plates together with the PCR mix. These reactions were transferred onto QuantStudio 12 K Flex OpenArray plates with the QuantStudio 12 K Flex AccuFill System (Thermo Fisher Scientific). Genotyping assays were run in a QuantStudio 12 K Flex real-time PCR instrument (Thermo Fisher Scientific) in standalone mode. Primers and probes are indicated in Supplementary Table S2. Nucleotide and haplotype diversities were calculated with the DnaSP software v5^14^. The Network software v5 (www.fluxus-engineering.com) was used to construct a median-joining network with default parameters^15^.

**Table 2:** Y-chromosome polymorphisms and haplotypes analyzed in the current work.

SRY 2971	SRY 3098	SRY 1876	AMELY 42	ZFY 527	ZFY 46	DDX3Y 56	Haplotype
T	G	A	C	A	C	G	Y1A
A	G	A	C	A	C	G	Y1B1
A	G	A	C	A	T	G	Y1B2
A	G	C	C	A	C	G	Y1C
T	A	A	C	G	C	C	Y2

## Results and Discussion

**Figure 1:**
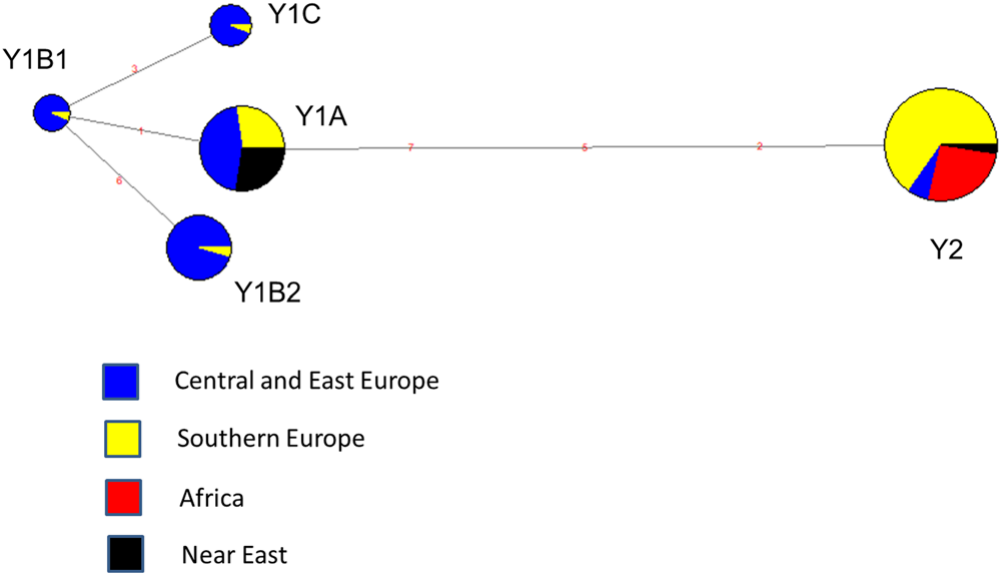
Median joining network based on the Y-chromosome genotypes of 275 goats from Southern European (Bermeya, Blanca Andaluza, Garganica, Guadarrama, Malagueña, Maltese, Murciano Granadina, Mallorquina, Pitiüsa, Blanca de Rasquera and Sarda breeds), Central and East European (Alpine, Appenzell, Camosciata delle Alpi, Chamois Coloured, Grisons Striped, Peacock Goat, Carpathian, Saanen, St Gallen Booted goat, Toggenburg, Valais Blackneck and Verzasca breeds), African (Djallonke, African Dwarf, Nigeria, Sahel and Zaraibi breeds) and Near Eastern (Iran and Oman) countries.

Sequence alignment made possible to identify four unreported polymorphisms in the non-coding regions of the genes *AMELY* (g.310_313delATAT in Genbank MF448227-MF448228), *DDX3Y* (g.56 C > G in MF448229-MF448230 and g.390 T > C in MF448231-MF448232) and *ZFY* (g.46 C > T in MF448233-MF448234). The deletion detected in the gene *AMELY* and the polymorphism in the intron 7 of the DDX3Y gene were discarded from the genotyping panel due to technical reasons. We therefore aimed to genotype five Y-chromosome SNPs (*SRY*-2971T > A, *SRY*-3098G > A, *SRY*-1876A > C –GenBank D82963-, *AMELY*-42C > T –GenBank AY082491.1-, and *ZFY*-527A > G –GenBank AY082500) that had been reported in previous studies^7–10^ plus two SNPs discovered by us (*DDX3Y* g.56 C > G and *ZFY* g.46 C > T). Altogether, these mutations defined five haplotypes *i.e*. Y1A, Y1B1, Y1B2, Y1C and Y2A (Table 2) whose frequencies were estimated in 275 bucks from 31 populations (Table 1). In general, the diversity of the caprine Y-chromosome was quite low (2.1 polymorphisms/kb, Table 3), a result that matches previous data reported in sheep^16^. These findings might be explained by the low effective size of the Y-chromosome mainly as a consequence of the high variance in reproductive success of bucks and rams. Another important factor that decreases Y-chromosome variation is the removal of deleterious mutations by purifying selection^17^. Due to the non-recombining nature of the Y-chromosome, this type of selection can erase variability even at linked neutral sites located far away from the purged mutation^17^. Interestingly, the analysis of Y-chromosome variation in pigs has uncovered the existence of a remarkable level of polymorphism^18^. This discrepancy between goats and pigs regarding the amount of paternal variation could be due to the fact that pigs were independently domesticated at two distinct sites and on the basis of two gene pools (Asia and West) that diverged around 1 Ma ago. This resulted in the emergence of two main Y-chromosome haplogroups that are highly differentiated^18^. In strong contrast, all modern goats descend from bezoars domesticated at Eastern Anatolia^11^ and this is probably the main reason why all Y-chromosome haplotypes are remarkably similar. The median joining network displayed in Fig. 1 shows that the distribution of Y-chromosome diversity is tightly linked to geography. In goats from Switzerland, the Y1A and Y1B2 haplotypes are clearly predominant (67%), while Y1B1 (15%) and Y1C (18%) are less frequent and Y2A cannot be found. In stark contrast, the most frequent haplotype in Italian and Spanish goats is Y2A (77%) followed by Y1A (18%), whilst frequencies of Y1B and Y1C are almost negligible. Interestingly, the Y2A haplotype is the only one segregating in African breeds. In a previous study, Pereira *et al*.^12^ analyzed the Y-chromosome diversity of goats from Morocco and Portugal and also found that the most abundant haplotype in both populations is Y2, though Y1A and Y1B haplotypes could be also detected. In goats from Iran, we have found that Y1A is the most frequent haplotype (90%). In contrast, Çinar-Kul *et al*.^8^ demonstrated that in Turkey the Y2A haplotype is predominant with the exception of a single breed, Kilis, where Y1A segregates at high frequencies. The coexistence of several differentiated Y-chromosome haplotypes in this area is consistent with the high diversity that is usually found in primary domestication centers.

**Table 3:** Caprine Y-chromosome diversity in four geographic areas.

Geographic areas	Number of haplotypes	Haplotype diversity	Sample size
Central and East Europe	5	0.747	113
Southern Europe	5	0.369	106
Near East	2	0.273	23
Africa	1	0.000	33
TOTAL	5	0.690	275

Central European breeds analysed in our study come exclusively from Switzerland. The analysis of autosomal variation in 10 Swiss breeds has shown that several of them (Appenzell, Toggenburg, Valais and Booted goat) display low levels of diversity but in general long runs of homozygosity (>15 Mb) are rare indicating the absence of recent inbreeding^19^. At this point, we do not know if Y-chromosome data obtained by us in Swiss populations can be extrapolated to other breeds from Central or North Europe. However, it is worth to emphasize that the patterns observed by Lenstra^7^ in North Central European breeds (*i.e*. Y1 haplotypes are abundant and Y2 is scarce) closely match those observed by us in Swiss populations. Significant genetic differences between Central and Southern European breeds have been also observed at the autosomal level. In this way, Cañón *et al*.^20^ genotyped a panel of microsatellites in 1,426 goats from 45 European and Near Eastern breeds and showed that Mediterranean and Central European breeds are clearly differentiated. This marked genetic differentiation could be due to several reasons. The dispersal of goats from the domestication center at Eastern Anatolia into Europe followed two main routes *i.e*. the Mediterranean and the Danubian corridors^21^. The Danubian route involved the transportation of goats through the continental heartland of Europe towards the Danube Valley and the central and northern plains of Europe^21^. Pastoralist communities were established in Greece and Bulgary 6,500 YBP and they subsequently moved north and eastwards until arriving to Scandinavia and the British Isles 4,000 YBP^22^. In stark contrast, the dissemination of domestic animals and plants through the Mediterranean corridor was essentially maritime^23^, with Neolithic seafarers reaching the Iberian Peninsula 7,700-7,300 YBP and Libya and Algeria 7,000 YBP. Thus, it is possible that the two Eastern Anatolian goat gene pools that followed the Danubian and Mediterranean corridors were subjected to serial founder effects resulting in the genetic differentiation of Swiss and Italian/Spanish European goat breeds. Indeed, the results of Çinar Kul *et al*.^8^ indicate that, in Turkish goat breeds, the Y1A haplotype is almost fixed in Kilis bucks, while Y2A nearly reached fixation in Abaza, Gurcu, Angora and Norduz bucks. This genetic heterogeneity of Turkish breeds would fit a scenario based on the post-domestication spread of different gene pools along the Danubian and Mediterranean corridors.

An alternative, but not excluding, explanation for this pattern of differentiation would be the existence of gene flow between Southern European and North African goat populations. Pereira *et al*.^12^ hypothesized that the similar frequencies of Y-haplotypes that they observed in Portuguese and Moroccan goats might be due to the occurrence of ancient genetic exchanges. Martínez *et al*.^24^ shed light on this issue by demonstrating, with the aid of coalescent genealogy samplers, the existence of a significant and bidirectional migration between northwest African and Iberian goat populations. In a subsequent study, Manunza *et al*.^25^ analysed the variation of Spanish and African breeds on the basis of 52,000 SNP genotypes and found evidences of admixture between Andalusian (Murciano-Granadina and Malagueña) and Tunisian goats. In a recent study, Decker *et al*.^26^ showed that Spanish cattle breeds had a 7.5–20% of African introgression into their genomes, and an indicine introgression was also observed in Italian breeds. Taken together, these results suggest that admixture events may have contributed to the genetic differentiation of Central and Southern European goats.

Particularly intriguing is the case of Carpathian bucks from Romania which displayed high frequencies (42%) of the Y2A haplotype. This might likely be the consequence of the historical commercial relationships between this region and Turkey, or even the arrival and establishment, during the 8–12^th^ centuries, of Turkic nomad peoples (*e.g*. pechenegs and cumans) who migrated from the Central Asian steppes to Romania, very likely carrying their own livestock. Lack of arable land, drought and harsh and cold winters explain why nomadic pastoralism has been so prevalent in the Eurasian steppe since prehistoric times. Indeed, the analysis of sheep mitogenomes has revealed the existence of remarkable levels of genetic diversity in the Mongolian Plateau, suggesting that this region constituted one of the main centers of sheep dispersal across Asia^27^. Even nowadays, pastoral nomadism is the main form of land use in Mongolia, with one third of the population living as nomads from livestock breeding^28^.

Data presented by us and others^7–9^ indicate that Y-chromosome markers recapitulate much better the population structure of goat breeds than mitochondrial polymorphisms^29^. However, this does not mean that the maternal and paternal histories of goats are substantially different. The larger physical size of the Y-chromosome, if compared with the mitochondrial genome, facilitates the detection of more ancient drift signals^30^. Previous data based on microsatellite^24^ and SNP^25^ markers have revealed that Spanish goats are more closely related to their Central European counterparts than to the African ones, an outcome that does not fully match our analysis of Y-chromosome markers. The large scale study of genome-wide data from caprine breeds with a worldwide distribution will probably shed light on this and other issues that are essential to understand the history of goat pastoralism.

### Ethics statement

Blood and hair root samples were collected from goats by trained veterinarians in the context of sanitation campaigns and parentage controls not directly related with our research project. For this reason, permission from the Universitat Autònoma de Barcelona Committee of Ethics in Animal Experimentation was not required. In all instances, veterinarians followed standard procedures and relevant national guidelines to ensure appropriate animal care.

## Electronic supplementary material

Supplementary Tables S1 and S2
