# Supplementary material for: Differential distribution of Y-chromosome haplotypes in Swiss and Southern European goat breeds
Source: Sci Rep. 2017 Nov 23;7:16161. doi: 10.1038/s41598-017-15593-1 (PMC5701018; doi:10.1038/s41598-017-15593-1)
Supplement: Supplementary file 1 — Supplementary Tables S1 and S2 [file 41598_2017_15593_MOESM1_ESM.pdf]

Gene	Primer	Sequence	Location	Previous sequences:<br>Species (Sequence ID)	Goat regions GeneBank ID
AMELY	AMELYF	ACTTACACCTCTGAAGTGGTAT	Exon 3	*Ovis aries* (AY604731), *Bos taurus* (ENSBTAG00000047774)	MF448227, MF448228
	AMELYR	CCATCCACCAACGGGTTCATAG	Exon 4		
DBY	DBY1F	ACACTGGCACTTGAATGTTG	3'UTR	*Ovis aries* (AY604732), *Capra hircus* (XM_018044671)	MF448229, MF448230
	DBY1R	CCTTGGCATGTTATAGTTTC	3'UTR		
	DBY2F	ATGGGRAACATTGARCTTAC	Exon 7	*Bos taurus* (ENSBTAG00000045750), *Homo sapiens* (NM_004660), *Sus scrofa* (KU705630), *Canis lupus* (JX964855)	MF448231, MF448232
	DBY2R	GGACCATCTGWATAAATCTGAC	Exon 8		
UTY	UTY1F	ACCGAGAAGCAAGACTAAGGAAG	5'UTR	*Ovis aries* (AY604736), *Bos taurus* (ENSBTAG00000046123)	MF448235
	UTY1R	GCCATCTTCGTGAAGGTTCAG	Intron 1		
ZFY	ZFYF	CAGGAAATGGATGACAGTG	Exon 5	*Ovis aries* (SEG_AY604737S), *Bos taurus* (ENSBTAG00000007730)	MF448233, MF448234
	ZFYR	AGCACTTGCAGTGCCACTCCGA	Exon 6		

Supplementary Table S1. List of primers employed in the amplification and sequencing of Y-chromosome genes

**SNP**	Assay	Forward Primer Seq.	Reverse Primer Seq.	Reporter 1 Sequence	Reporter 2 Sequence
AMELY-42C>T	AMELY2	TGCAGCCTCATCACCACATC	GGAGTGTTGGCCAGGAACTG	ACAGGCTGCTGAGCTG	CAGGCTGCTAAGCTG
DDX3Y-56C>G	DBYUTR	GAATGTTGAATGTCATCGTATATTACATAA	CTGGGTTTGCTTAGCTTGACTAAAA	AAGCTCACAGTAACCTG	AAGCTCACACTAACCTG
SRY-2971T>A	SRY1_1	GATTGGCCATTAGTTAGATGGTAGCATAT	GTGAGTGTTTCTGAAAGTACTATAGTTTGGA	CACTATAGACAATAAAACTTT	CACTATAGACAATATAACTTT
SRY-3098G>A	SRY1_2	TGGATGGAAAGCTCCCTACCTT	GGTGGAAAGTAGCTAGAAAGACCAA	AAGCACTTTCTGATACAAG	AAGCACTTTCTGGTACAAG
SRY-1876A>C	SRY2	GGCTGCCAGGAGGTATTGAG	GCTGTTCTTACCAGAGACTGACTT	TTCACTGCAAATTTT	CACTGCCAATTTT
ZFY-527A>G	ZFY1	TTGAGGTTTATGAATGTTTTCCCCAGAT	TGGTCCAGAAAACTCAAAATGACTGTAT	ATTGAAAGCCATACTTGCT	AAAGCCACACTTGCT
ZFY 46C>T	ZFY2	AGGTTTCATTGTCATAGGTTTCAGAAATATGA	GCATAAAGTAAGTTCCTATTAAAGTTAAAGACAAATGA	CAGTTTTTACTTAAATCAGTTCAC	TTTTTACTTAAATCAATTCAC

Supplementary Table S2. Primers and reporters used in the genotyping assays of the Y-chromosome polymorphic positions.
